# Towards a European health information system: The BRIDGE Health, InfAct and PHIRI projects

**DOI:** 10.25646/12943

**Published:** 2024-12-11

**Authors:** Martin Thißen, Angela Fehr, Aline Anton, Stefanie Seeling, Thomas Ziese

**Affiliations:** 1 Robert Koch Institute, Department of Epidemiology and Health Monitoring, Berlin, Germany; 2 Robert Koch Institute, Centre for International Health Protection, Berlin, Germany

**Keywords:** Non-communicable diseases, Health information, Health information systems (HIS), Europe, Indicators

## Abstract

**Background:**

National health systems in Europe are facing similar challenges – demographic change, a rising burden of disease due to chronic non-communicable diseases, and health inequalities. Comparable health data and knowledge sharing between countries are therefore an important basis for policy decision-making. However, health information in the European Union (EU) is fragmented and approaches to establishing a comprehensive system are largely project-based.

**Methods:**

This contribution describes the European projects BRIDGE Health (2015 – 2017), InfAct (Information for Action, 2018 – 2021) and PHIRI (Population Health Information Research Infrastructure, 2020 – 2023), which aimed at developing a sustainable health information infrastructure.

**Results:**

The projects, which build on one another, laid the groundwork for an EU-wide health information system. For example, a health information portal was established, a federated research infrastructure was implemented, handouts were created and training on capacity building was provided.

**Conclusions:**

An integrated EU-wide health information system is an important basis for policy decision-making and a prerequisite for a rapid and coordinated response to health crises. A sustainable structure or institution with a mandate for non-communicable diseases (NCD) and their determinants at the EU level would be desirable.

## 1. Introduction

### 1.1 What is health information and what are health information systems?

The health information systems (HIS) of European countries differ as a result of specific historical and cultural contexts [1]. Nonetheless, European countries are faced with similar demographic, health and health system-related challenges. They are confronted with ageing populations, a rising burden of disease, persistent inequalities and, therefore, growing pressure on their health systems [2].

Reliable health information is crucial for the development of strategies and concepts to meet these challenges and promote the health of people in all age and population groups. Such information includes data on health status, health determinants and the performance of health systems, enabling targeted research and supporting policy decision-making processes. Health reporting as a component of public health serves to provide timely, thematically relevant and high-quality health information [3].

**Figure 1:**
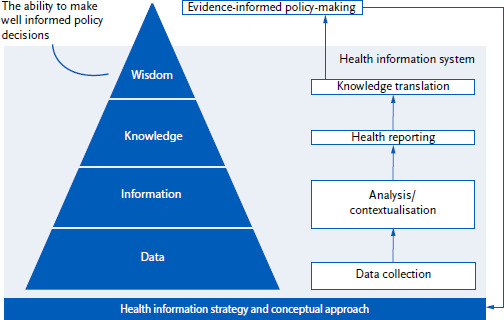
Information pyramid. Source: Adapted according to [4]

The information pyramid is a concept that is frequently used to illustrate various types or development phases of health information in the context of population-wide health monitoring (Figure 1). Firstly, a conceptual and strategic process takes place to select topics that are relevant to population health. Building on this, a national HIS provides the necessary infrastructure for the regular and timely performance of all steps in the monitoring cycle. Beside data collection, analysis and synthesis, reporting and knowledge transfer, it constitutes all resources, actors, activities and results that enable evidence-based health policy. HIS make it possible to strengthen research and monitoring in the area of public health and thus support evidence-based decision-making [4, 5].

### 1.2 European initiatives towards a European health information system

Health information is an essential building block in the development of a health system but is often fragmented and difficult to access in the European Union (EU). Insufficient data bases, differently defined indicators or varying survey methods result in a lack of comparability between and within EU member states [5, 6]. At the same time, it is important to be able to compare the health situation, health behaviour and health care of people in the EU Member States in order to support policy decision-making and strengthen health programmes. This can be illustrated using the example of demographic change. The significant rise in the share of elderly people entails an increase in chronic diseases and functional limitations among the population and requires an appropriate adaptation of health systems in order to ensure adequate care and nursing for affected individuals [7].

**Key statements**

► Non-communicable diseases are the leading cause of premature death in Europe.
► Resilient health systems and the promotion of Health in all Policies require evidence and scientific exchange between Member States.
► Data must be processed and communicated in a targeted manner in order to strengthen health literacy among the population.
► Europe needs a permanent structure now for the collection, analysis and communication of health information to jointly manage shared health challenges in non-communicable diseases and their determinants.

Both the European Council and the European Commission seek an improved alignment of health information activities at the EU level in terms of coherence, coordination and sustainability; data harmonisation, collection, processing and reporting; research, capacity building and transferability into evidence-based policy. Since the late 1990s, with the development of the European Core Health Indicators (ECHI) [8], European research networks have been established by merging EU-funded projects in order to strengthen evidence-based health policy and research. As a result of EU indicator projects, the European Health Interview Survey (EHIS) was created in 2006 as a now mandatory instrument to collect health data in the EU. As the statistical authority of the EU, Eurostat (ESTAT) makes data from this and other health-related surveys available for the purpose of policy and research. Furthermore, the Expert Group on Health Information (EGHI) has been advising the EU Commission on technical and strategic matters concerning the use and further development of health information in the EU since 2009 [9]. The BRIDGE Health (BRidging Information and Data Generation for Evidence-based Health policy and research, 2015 – 2017), InfAct (Information for Action, 2018 – 2021) and PHIRI (Population Health Information Research Infrastructure, 2020 – 2023) projects paved the way for building a sustainable health information infrastructure in the EU.

**Relevant websites**

BRIDGE Health

https://www.bridge-health.eu/

InfAct

https://www.inf-act.eu/

PHIRI

https://www.phiri.eu/

European Health Information Portal

https://www.healthinformationportal.eu/

### 1.3 Objective and structure of the contribution

The objective of this contribution is to a draw a link between the successive EU projects for the development of a sustainable health information system and to demonstrate what successes have been achieved, what challenges have been identified and what prerequisites are necessary for the sustainable implementation of an EU HIS. To this end, the following remarks will present the individual initiatives, their content and results and, lastly, discuss them with a view to the future.

## 2. Description and objectives of the projects

**Figure 2:**
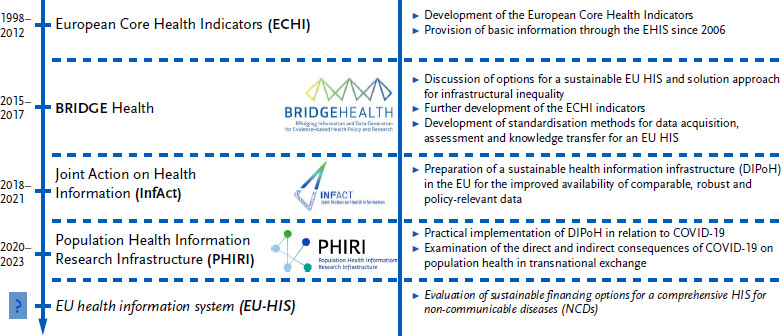
Progressive objectives of the BRIDGE Health, InfAct and PHIRI projects

Figure 2 shows the goals of the three EU-funded projects BRIDGE Health, InfAct and PHIRI, which successively build on one another and are based on the development of the ECHI set of indicators.

### 2.1 BRIDGE Health

BRIDGE Health was, in the literal sense, a bridge project. After four successive EU-funded projects (ECHI-1, ECHI-2, ECHIM and Joint Action ECHIM) between 1998 and 2012 to develop and implement core health indicators, a financing gap threatened the long-term objective of building a European health information system. The EU closed this gap with funding for the BRIDGE Health project from 2015 to 2017. The temporary funding made it possible to continue the conceptual work on a European health information system and maintain the necessary interdisciplinary expert networks. BRIDGE Health comprised 31 partner organisations in 16 countries and bridged EU projects in the domains of health monitoring, health systems research, indicator development, health interview and health examination surveys, environment and health, accidents and injuries, disease registries, and clinical and administrative health data. Besides the thematic work packages that resulted from these EU projects (e.g. the inclusion of indicators for environmental health, health information systems, and the health of mothers, children and adolescents), another particularity of the project structure was the identification of cross-cutting issues (e.g. data quality, standardisation of data acquisition, reduction of inequalities in the availability of health information or the use of scientific findings for policy decision-making), which purposefully integrated contributions from the work packages. On the whole, the thematic BRIDGE-Health work packages dealt with the concept of a European health information system and the required indicators and data acquisitions, while the cross-cutting issues primarily expanded on methodological approaches.

The overarching objective of the project was to reduce health inequalities between and within the EU Member States. This can only be achieved through the improved availability of valid population-wide health data. These data provide the information required for the development and implementation of targeted prevention and care structures. For this purpose, among other things, the Robert Koch Institute (RKI) conducted a survey on the state of data availability for the European Core Health Indicators (ECHI) in the participating countries. Furthermore, BRIDGE Health was intended to break the cycle of temporary projects and develop a concept for a sustainable European health information system (EU HIS). BRIDGE Health thus marked the transition from projects focussed on data and indicators to the establishment of a European cooperation for the development of an EU HIS.

### 2.2 InfAct

The conceptual and technical work begun by BRIDGE Health was continued and intensified in the immediately subsequent co-funded Joint Action on Health Information (InfAct). Joint Actions are a funding model in which the EU Commission and Member States contribute to the financing of a project according to a fixed ratio. InfAct ran from 2018 to 2021 and included 40 partner organisations from 28 countries. The project comprised ten work packages, three of which dealt with project coordination, internal evaluation and the dissemination of project results. Two work packages had a strategic focus: these further developed the concept for a European health information system and prepared a roadmap for the sustainable use of the project results at the national and European level. Five content-related work packages involved conducting assessments of health information systems in selected project countries, creating extensive overviews of the structure and quality of national health reporting, identifying training needs and developing action plans for standardising the provision of health data within the EU. Additionally, proposed solutions to legal, organisational, semantic and technical interoperability challenges were developed. Semantic challenges arise, for example, in the cross-border harmonisation of data that has been collected at the national level due to differently defined indicators or varying indicator lists. Coding according to the International Classification of Diseases, by contrast, contributes to semantic interoperability, for example. Furthermore, the application of the FAIR principles is recommended, according to which data should be ‘Findable, Accessible, Interoperable and Re-usable’.

### 2.3 PHIRI

Based on the concept for a European health information system for population health developed during BRIDGE Health and InfAct, the subsequent Population Health Information Research Infrastructure (PHIRI) project served as a practice-oriented pilot study using the example of COVID-19. Through a close collaboration with 41 partner institutions from 30 countries, PHIRI aimed to provide powerful research tools and services to strengthen collective knowledge [10]. PHIRI essentially consisted of three pillars:

1. *Exchange of health data, information and knowledge.* The European Health Information Portal serves as a one-stop shop facilitating the provision of population health and health care data, information and expertise in Europe.
2. *Research and innovation.* Research is also supported by the identification, availability, assessment and reuse of population health data and other health care data.
3. *Recommendations for policy actions.* When the COVID-19 pandemic broke out, the resilience of HIS in European countries varied greatly. PHIRI conducted COVID-19 assessments of national HIS in order to derive lessons for future pandemics and crises.

## 3. Results of the projects

Table 1 shows a breakdown of the outcomes resulting from BRIDGE Health, InfAct and PHIRI divided into the following categories:

► European Health Information Portal with catalogues of population health information sources from national and international organisations
► Federated research infrastructure, in which sensitive data remains with the data-holding institutions and only analytical scripts and aggregated results are exchanged through a coordinating node, with use cases and the secondary use of data in order to answer research questions
► Guidelines and tools for dealing with health information
► Strengthening health information literacy

### 3.1 BRIDGE Health

BRIDGE Health showed that the instruments and structures in place in Europe at the time were not capable of meeting the requirements for valid and timely population health information and derived measures without a European institutional framework. Examples of this are the list of European core indicators, the presentation of results by Eurostat, the EU Expert Group on Health Information and temporary scientific projects.

The recommendations (or demands) from the BRIDGE Health project were summarised in two final documents, which were addressed to national governments and the European Union. The first document [11] outlines the creation of a European Research Infrastructure Consortium on Health Information for Research and Evidence-based Policy as a solution to the determined infrastructural deficits and inequalities. Although the establishment of an institution similar to the European Centre for Disease Prevention and Control (ECDC) [12] was seen as the ideal solution, after consultations with stakeholders in the EU Member States, the project participants were of the opinion that the introduction of a research consortium was more feasible in the short to medium term. The separate legal personality of a consortium would make it possible to procure EU funding, and its administration by the Member States, including the collection of membership fees, would ensure the sustainability of the association.

A second document [13] summarised the scientific and technical tasks that should be performed by a research consortium for health information. The consortium would ensure the further development of the European Core Health Indicators in the long term and thus the necessary data acquisitions, data analyses and the creation of target group-appropriate information products for evidence-based policy. It should also play a significant role in the networking of European experts and as a knowledge platform. Drawing on experience with temporary projects and efforts to maintain expert networks within Europe beyond the end of the project, the consortium should enable and promote sustainable collaboration between experts, provide know-how, such as examples of good practice or methodological handouts, and offer researchers a point of contact for technical questions. The documents developed during BRIDGE Health included recommendations concerning health data quality assurance (especially in population-wide disease registers), ethical and legal issues, overviews of national data acquisition methods, methods for calculating healthy life years, and the interoperability of health data.

The BRIDGE Health project concluded with this overall concept for the structure and tasks of a planned EU HIS and with numerous technical reports on its future thematic focuses based on the individual work packages. The objective of moving away from a series of temporary projects and shifting to a sustainable infrastructure was not achieved with BRIDGE Health. Nevertheless, the project results formed the basis for the immediately subsequent project of a Joint Action on Health Information (InfAct).

### 3.2 InfAct

The most visible and sustainable outcome of the InfAct project is the European Health Information Portal [14] (see Infobox). This was initiated through a combination of multiple InfAct work packages in order to make project outcomes digitally retrievable as well as to provide long-term and dynamic access to information about data, research projects and actors in the field of European population health [15]. The portal was finalised as part of the follow-up project PHIRI. The portal is aimed at researchers and policymakers seeking information on health projects, health data and contact partners in Europe. Besides its own project results and training measures, the portal links, among other things, to the database of the European Core Health Indicators (ECHI data tool), national, European and international organisations, and completed and ongoing research collaborations.

Another key milestone of InfAct was the submission of a concept for a Distributed Infrastructure on Population Health (DIPoH) for the Roadmap 2021 of the European Strategy Forum on Research Infrastructures (ESFRI) [16]. DIPoH should establish a European infrastructure for population-based health information that supports open, networked and data-oriented health research throughout Europe. The European research infrastructures (European Research Infrastructure Consortium, ERIC) are research networks that are established and managed by member states and are financed by membership fees and third-party funding. To set up an ERIC, an application must be submitted and the project is subject to a selection procedure by ESFRI, a multidisciplinary forum to promote the scientific integration of European countries. The first hurdle for proposed European cooperation projects is inclusion in the ESFRI Roadmap; this is a prerequisite for participation in the selection procedure. The submission deadline during the project duration fell in May 2020. In June 2021, the project leaders learned that the application was unsuccessful and that the DiPoH would not receive any funding via the ESFRI Roadmap 2021 [17]. Although the concept was recognised as being of high pan-European relevance, deficits were identified in terms of the communication strategy, the planned access to data, and the organisational structure.

The project work had progressed in the meantime, and the sustainability and further development of the project results had been ensured with the European Health Information Portal. An example of this are the National Nodes (NNs). Often overseen by the participating national public health institutes, these nodes are national contact partners and points of contact for cooperation requests between EU Member States on topics related to population health. The European Health Information Portal provides access to the NNs. Additionally, the portal delivers an overview of European research projects and networks and links to relevant international and national organisations, such as the NCD Alliance for non-communicable diseases or the World Diabetes Foundation. The portal provides complete information on the development and implementation of the European Core Health Indicators (ECHI), including examples of their application at the national level as well as the ECHI database with current survey data. It also provides access to the results and methods that InfAct developed for assessing national health information systems. Building on methods of the World Health Organization Regional Office for Europe (WHO/Europe) a peer-to-peer assessment method was developed and piloted in nine project countries, and the adapted methodology was published for further use. Peer-to-peer assessments and situation analyses of health information systems with a focus on non-communicable diseases (NCDs) using this method were applied in further EU projects during the COVID-19 pandemic (see 3.3). As a further outcome, the portal contains training offers in the area of health information; these were primarily expanded during the COVID-19 pandemic (see 3.3). In addition, InfAct developed a guideline on the availability and quality of health information and collated examples of new indicators and data sources for priority health topics.

Despite extensive lobbying by the project participants, there was a lack of political will in the EU and in several member states to create a sustainable European health information system during the BRIDGE Health and InfAct projects. Further activities in this direction were subsequently temporarily overshadowed by the COVID-19 pandemic. This presented an opportunity to use the infrastructures, methods and expert networks established during BRIDGE Health and InfAct to implement specific projects that were relevant to the COVID-19 response for the protection of population health. The subsequent EU project PHIRI, which built on InfAct, arose out of the necessity to also consider non-communicable diseases and the stability of health care provision in the pandemic response.

### 3.3 PHIRI

Building on the preliminary work of InfAct, one of the key elements of PHIRI involved the development and testing of a federated research infrastructure on the basis of four use cases that examined the impacts of the COVID-19 pandemic on specific subgroups. For this purpose, routine data (patient records), administrative data and research data were used and combined with contextual data at the population level. The four use cases, the results of which can be found in the listed references, considered:

- Direct and indirect determinants of the COVID-19 pandemic in vulnerable population groups with reference to inequalities [18].
- COVID-19 related delayed treatment of breast cancer patients [19].
- The impact of COVID-19 on perinatal health inequalities [20].
- Changes in population mental health [21].

**Figure 3:**
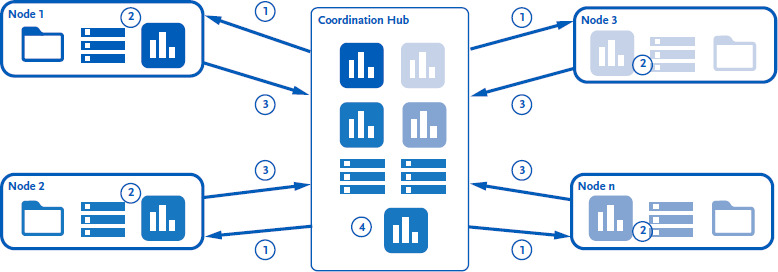
Architecture of the federated data analysis in PHIRI. Source: [23]

The process of data analysis in PHIRI can be considered as a solution for a federated data network. This aims to facilitate access to data without undermining data security or privacy law. In the federated research infrastructure, the data sets never leave the data-holding institutions (data hubs), and only analytical scripts and aggregated results are exchanged (see Figure 3). The data hubs specify the research question using the central coordinating node (coordination hub) in a common data model (CDM). On this basis, the coordination hub prepares the analytical pipeline (e.g. scripts for the statistical software R) and distributes the corresponding results to the data hubs (step 1) in a web-based application, a Docker image [22]. The data hubs run the analyses locally (step 2) and transmit the aggregated results back (step 3). The ‘local results’ are aggregated and reused for joint and comparative analyses [23]. In total, nine data hubs and 28 members of the Euro-Peristat Network [24] conducted analyses in this way and published the results [18–21].

Using the National Nodes (NNs) identified during InfAct, PHIRI was able to publish a health information portal with references to data sources, population health studies, training materials and courses, whereby ethical and legal aspects were taken into account (see 3.2) [15]. In addition, PHIRI developed a framework for assessing the direct and indirect impacts of COVID-19 on population well-being, morbidity and mortality [25]. The established Rapid Exchange Forum (REF) served as a bi-weekly exchange meeting and provided rapid answers to research and policy questions related to tackling the COVID-19 pandemic. The questions were posed in the participating EU Member States as well as by international organisations, such as WHO, the Organisation for Economic Co-operation and Development (OECD) and the Joint Research Centre (JRC) of the European Commission. The exchange format of the REF continues to be implemented in the broader context of population health beyond the end of the project [26]. National health information system assessments regarding the effects of COVID-19 on population health in eight European countries were carried out on the basis of the preliminary work from InfAct (see 3.2) [27]. Lastly, the development of methods to model scenarios for national situations was advanced in order to gain insight into possible future health impacts of the COVID-19 outbreak [28]. The extent to which the results of the PHIRI project – as a form of practical lobbying, in a sense – have led to greater political support for an EU health information system for non-communicable diseases remains to be seen.

## 4. Discussion

Transparency, clear communication and active public participation help increase the population’s trust in governments and public institutions. The availability and trustworthiness of population health data are vital, not least in times of crisis, such as during the COVID-19 pandemic [29]. Especially in the area of population health, a structured European mechanism is urgently needed in order to organise and exchange high-quality health information between countries.

Policy-relevant analyses generally comprise four elements, which can also be visualised in a HIS: Trends (progress), trajectories (positive or negative changes), triggers (thresholds for the implementation of measures) and targets (desired indicator levels). Ideally, a European HIS encompasses both health care data and data from population-based surveys. In summary, they make it possible to monitor access, quality and equal opportunity with regard to health care provision as well as trends and developments in health determinants. A HIS could also be a suitable infrastructure for the integration and analysis of large volumes of NCD-relevant data (big data analysis) that provide policymakers with up-to-date information [30].

The BRIDGE Health, InfAct and PHIRI research projects are important building blocks on the way towards a HIS at the European level. The consortium of interdisciplinary experts from the EU Member States in the fields of health information and population health, which has been built up over many years, has identified needs for a sustainable infrastructure for health information in the EU and laid the foundation for a transnational HIS. Beyond long-term strategic considerations, each project also delivered up-to-date scientific findings.

The European Health Information Portal established as part of the projects represents an important milestone on the way towards an EU-wide HIS. It provides a central entry point and a knowledge base for researchers, policymakers and other relevant interest groups. The provision of tools, such as examples of good practise or methodological handouts, as well as the implementation of training programmes in the form of the European School on Health Information to promote capacity building and knowledge exchange [31] form the basis for the transnational organisation and exchange of high-quality health information. All this content can be accessed via the portal [14]. Sufficient resources are required to ensure the sustainability of the platform. Besides maintaining existing content, new sections and functions should also be added in order to continuously meet evolving information needs. The National Nodes are therefore also needed beyond the project duration in order to keep content up to date and respond to future challenges. Within the PHIRI consortium, meetings have been and will continue to be organised with the goal of improving the management and content of the European Health Information Portal. A user survey could provide further insight into the needs of target groups and opportunities for the development of the portal [15].

Building on the results of the preceding BRIDGE Health and InfAct projects, PHIRI also formed an integral cornerstone for ongoing European initiatives. PHIRI provided an opportunity to put the prerequisites for a sustainable, federated population health research infrastructure to the test using the example of COVID-19 and thus promote national and European pandemic preparedness by improving the availability of comparable, reliable and policy-relevant data. In future, it will be necessary to make adjustments to the federated research infrastructure. Currently, the architecture of the federated data analysis requires a high level of human resources, especially due to the necessity of a central coordination hub. With the goal of being able to answer a larger volume of research questions, the processes should be designed so that interactions are supported by computer-based assessments (machine-to-machine) and individual data-holding institutions dynamically assume the role of coordination hub (peer-to-peer). A challenge lies in the fact that the IT capabilities of the data hubs in terms of system administration (e.g. using the Docker container) or analytical methods (e.g. using Python, R) are heterogeneous in the EU. Especially against this background, capacity building and knowledge exchange are of great importance for implementation beyond the research project [23].

The European Health Data Space (EHDS) initiated by the EU aims, among other things, to invigorate the European health data ecosystem through the provision of tools and services for the secondary use of health data [32]. It builds on the federated research infrastructure developed and successfully tested during InfAct and PHIRI in order to facilitate the reuse of data beyond national borders and consolidate strong networks and platforms. In future, this should provide increasing amounts of reliable and comparable health information for evidence-based policy decision-making processes [10].

Nevertheless, the EU still lacks a sustainable structure or institution for non-communicable diseases and their determinants along the lines of the ECDC for infectious diseases. Numerous surveillance and indicator systems for NCDs have been implemented within the framework of temporary projects. Although such projects generate targeted approaches, they do not provide a basis for a sustainable health information system. Filling this gap would require a permanently funded HIS that amalgamates EU health information. It would be desirable to provide health-related information at the data, indicator and knowledge levels through the creation of summary reports and assessments. This would improve the basis for developing suitable evidence-based measures in the public health system that aim to promote population health in the EU [33]. Building political will and achieving the goal of a European HIS will require the concerted efforts of all stakeholders in public health at the national and EU level.

## Figures and Tables

**Table 1:** Scientific results from BRIDGE Health, InfAct and PHIRI

Categories	BRIDGE Health (2015 – 2017)	InfAct (2018 – 2021)	PHIRI (2020 – 2023)
European Health Information Portal		Development of the health information portal Identification of routine population health data sources and possible contact persons in the EU Member States Metadata structure and access	Integration of: ► Data sources and publications ► International guidelines, initiatives and projects ► Training materials and courses ► Tools for dealing with ethical and legal issues ► Contact details of contact persons for health topics Activation of the European Health Information Portal
Federated research infrastructure	Identification of the need for a research infrastructure	Implementation and piloting of a federated research infrastructure Areas of application: ► Stroke care ► Indicator on resilience ► Cost of dementia care	Further development and piloting of the federated research infrastructure Areas of application: ► Vulnerable population groups ► Perinatal health ► Delayed cancer care ► Mental health
Guidelines and tools for health information	Identification of health data needs Recommendations for health data quality assurance Ethical and legal framework conditions for dealing with health data Overviews of national data acquisition methods Methods for calculating healthy life years Improving the interoperability of health data	Development of the ECHI short list Good practise for national health reporting Recommendations for dealing with and using machine learning techniques Guidelines for prioritising health information Evaluations of health information systems in selected project countries Tool kit to assist the burden of disease ‘ calculation Further development of the interoperability of health data	Bi-weekly exchange of expertise (Rapid Exchange Forum) on COVID-19 (continues to be implemented in the broader context of population health) Research methods for evaluating the effects of the COVID-19 pandemic Foresight: modelling and scenarios Evaluations of health information systems in selected project countries
Competence building		European School on Health Information: ► Privacy (General Data Protection Regulation, GDPR) ► Training on the implementation of the European Health Examination Survey (EHES) ► Burden of disease calculation ► Implementation of HIS assessments	European School on Health Information: ► Foresight methods for evaluating the broader impacts of COVID-19 ► Infodemic management ► Training for data owners ► Implementation of HIS assessments
